# Fusing wrist pulse and ECG data for enhanced identification of coronary heart disease and its complications

**DOI:** 10.3389/fphys.2025.1628309

**Published:** 2025-07-29

**Authors:** Lei-Xin Hong, Wen-Jie Wu, Xia Chen, Dan-Qun Xiong, Ye-Qing Zhang, Xiang-Dong Xu, Jian-Jun Yan, Rui Guo

**Affiliations:** ^1^School of Traditional Chinese Medicine, Shanghai University of Traditional Chinese Medicine, Shanghai, China; ^2^Department of Cardiology, Shanghai Jiading District Central Hospital, Shanghai, China; ^3^Department of Chinese Internal Medicine, Shanghai Municipal Hospital of Traditional Chinese Medicine, Shanghai, China; ^4^School of Mechanical and Power Engineering, Institute of Intelligent Perception and Diagnosis, East China University of Science and Technology, Shanghai, China

**Keywords:** coronary heart disease, complications, synchronous acquisition of ECG and PPW, machine learning algorithms, modeling

## Abstract

**Objectives:**

This study aimed to explore the potential of synchronously acquiring wrist pressure pulse wave (PPW) and limb lead electrocardiogram (ECG) signals for the development of an identification model for coronary heart disease (CHD) and its associated comorbidities.

**Methods:**

A custom-designed device equipped with pressure and ECG sensors, was utilized to synchronously collect wrist PPW and limb-lead ECG signals from 748 participants (463 for modeling and 285 for external validation). Features were extracted from these two types of physiological signals to form distinct datasets, and RF models were built based on different datasets. The top-performing RF model was then selected and compared against the Feature-Selected (FS-RF), Support Vector Machine (SVM) and Bagged Decision Tree (BDT) models. Ultimately, the optimal model for predicting coronary heart disease (CHD) and its comorbidity was determined based on evaluation metrics.

**Results:**

The RF model that integrated both PPW and ECG features demonstrated significantly higher effectiveness compared to the RF model that relied on a single physiological signal. Furthermore, when benchmarked against the feature-selected RF(FS-RF), SVM and DBT models, the FS-RF model demonstrated the best performance, achieving an accuracy of 76.32%, an average precision of 75.82%, an average recall of 76.11%, and an average F1-score of 75.88%, all of which were higher than those of other models. Notably, the selected feature by FS-RF encompassed both PPW and ECG features.

**Conclusion:**

This study highlights the importance of synchronously acquiring of PPW and ECG signal, along with feature selection, in enhancing the performance of the FS-RF model for identifying CHD and its associated conditions. These findings provide a scientific basis for the application of wearable devices in clinical settings, highlighting their potential to aid in the early detection and management of cardiovascular disease.

## 1 Introduction

Coronary heart disease (CHD) is a common chronic cardiovascular condition, primarily caused by coronary atherosclerosis, which leads to vascular lumen narrowing or occlusion, subsequently resulting in myocardial ischemia, hypoxia, and even infarction. Comorbidities such as hypertension and diabetes significantly increase the risk of cardiovascular events, severely affect patients’ quality of life, and place a substantial burden on healthcare systems. Despite ongoing medical advances, the global prevalence of CHD and its comorbidities remains high. Therefore, early identification and risk stratification remain pressing clinical challenges.

Pulse diagnosis, a traditional diagnostic technique in Traditional Chinese Medicine (TCM), involves physicians assessing a patient’s condition by palpating the wrist pulse with their fingers. With the rapid advancement of modern technology, signal analysis techniques have made significant progress, providing robust support fort the modernization of traditional medicine practices. Wrist pressure pulse wave (PPW) signal acquisition and analysis device has emerged as an objective and quantitative tool, capable of precisely capturing and analyzing pulse wave signals. This technology provides a powerful tool for disease classification and diagnosis. Compared to the traditional pulse diagnosis method, PPW signal analysis exhibits higher accuracy and reproducibility, aiding physicians in more scientifically assessing patients’ health status.

In recent years, remarkable achievements have been made in the medical field through the integration of PPW signals and machine learning methods. By employing sophisticated algorithms, researches have been able to extract latent information embedded within PPW signals, enabling precise disease classification and prediction. For example, Zhang et al. (2018) applied a three-class support vector machine (SVM) to distinguish PPW signals between healthy individuals and lung cancer patients, achieving an accuracy of 78.13%. Jiang et al. (2022) used sparse decomposition combined with an enhanced Gabor function to identify the PPW characteristic specific to diabetic patients, attaining an accuracy of 93.54%. Lyu et al. (2024) employed various machine learning classifiers to analyze PPW signals, with the Extra Trees classifier achieving an accuracy of 85.79% in classifying healthy individuals, CHD patients, and those with hypertension. Our research team has also contribution to this body of work by demonstrating the potential of PPW signals in assessing cardiac function (Wu et al., 2023; Zhang et al., 2024). Collectively, these findings underscore the value of integrating PPW signals with machine learning techniques for advancing medical diagnostics.

However, most existing studies predominantly rely on single-modality physiological signals, which are limited in their ability to reflect complex pathological processes. Consequently, multimodal fusion technologies have increasingly become a research focus. Wang et al. (2023) combined Electrocardiogram (ECG) and Photoplethysmogram (PPG) signals using a fusion matrix model to estimate blood pressure, achieving correlation coefficients of 0.988 and 0.991 for systolic and diastolic pressure, respectively. In a separate study, our team (Xiaotian et al., 2024) utilized pressure and photoelectric sensors to capture PPW and figure PPG signals, developing a random forest model that achieved 78.79% accuracy in assessing the severity of coronary artery disease. These studies highlight the advantages of multimodal approaches in enhancing diagnostic accuracy and reliability.

Despite these advancements, research on the synchronous acquisition and fusion analysis of ECG and PPW signals remains limited. ECG reflects the heart’s electrical activity, while PPW represents its mechanical function. The integration of these two modalities may offer a more comprehensive evaluation of cardiovascular status.

To address this research gap, our team has developed a novel device capable of synchronously acquiring ECG and PPW signals. In this study, we extract multimodal features of ECG and PPW signals from patients with CHD and related comorbidities, construct random forest (RF) classification models, and compare their performance with Support Vector Machine (SVM), Bagged Decision Trees (BDT), and feature-selected RF models. The results demonstrate that multimodal fusion, combined with feature selection, can significantly enhance the identification accuracy of CHD and related diseases, offering a promising avenue for improving diagnostic capabilities in clinical settings.

## 2 Data and methods

### 2.1 Participants

Participants were recruited from cardiology inpatients and individuals undergoing routine health assessments at the Physical Examination Center. The study was conducted over a two-year period, spanning from March 2021 to March 2023, at Yueyang Hospital of Integrated Chinese and Western Medicine, Shanghai Jiading District Central Hospital, Shuguang East Hospital and Shanghai Municipal Hospital of Tradition Chinese Medicine, all of which are affiliated institution of Shanghai University of Traditional Chinese Medicine.

The study population for modeling comprised 75 individuals diagnosed with CHD, 134 individuals with both CHD and hypertension, 102 individuals with a combination of CHD, hypertension and diabetes, and 152 healthy controls. For the purpose of analysis, participants were categorized into four distinct groups: Group 1 (healthy controls), Group 2(CHD patients), Group3 (CHD patients with hypertension), and Group 4(CHD patients with hypertension and diabetes). An additional 285 cases were for external validation. All data were obtained with written informed consent from the participants and were maintained under strict confidentiality protocols.

### 2.2 Diagnostic criteria

CHD diagnosis followed the ACC/AHA 2023 Guidelines (Virani et al., 2023), confirmed by coronary angiography (≥50% stenosis) or documented myocardial infarction. Hypertension was defined per Chinese Hypertension Prevention and Treatment Guidelines (2024 Revision) (Revision Committee of Chinese Guidelines for the Prevention and Treatment of Hypertension et al., 2024) as: systolic/diastolic blood pressure ≥140/90 mmHg on ≥3 separate days or current antihypertensive treatment. Type 2 diabetes mellitus diagnosis adhered to Chinese Type 2 Diabetes Prevention and Treatment Guidelines (2017) (Chinese Diabetes Society, 2018), requiring fasting plasma glucose ≥7.0 mmol/L and/or HbA1c ≥ 6.5%, or previously confirmed diagnosis with ongoing therapy.

All comorbidities and medications were triple-verified through: (1) hospital EHR-documented discharge diagnoses; (2) laboratory test reports within 6 months (including lipid profiles, glucose tests); (3) independent review by two cardiologists. Self-reported data conflicting with medical documentation were corrected per clinical records.

### 2.3 Inclusion and exclusion criteria

#### 2.3.1 Inclusion criteria

(i) Participants must fulfill aforementioned diagnostic criteria for the targeted diseases. (ii) Participants must be in good mental health, with no past history of severe mental disorders, and demonstrate the ability to fully cooperate with study procedures, including the collection process of clinical data. (iii) Participant must be aged between 20 and 75 years (iv) Complete general information and clinical data must be available for each participant. (v)Prior to participation, written informed consent must be obtained from all participants.

#### 2.3.2 Exclusion criteria

(i) Patients experiencing acute myocardial infarction during the study period or with a history of acute myocardial infarction within the past 3 months; those with acute heart failure, severe valvular diseases, pulmonary embolism, malignant tumors, mental disorders, or severe respiratory diseases. (ii) Individuals participating in a clinical trial or who have undergone significant therapy within the past 6 months (iii) Individuals with incomplete general information and clinical data.

### 2.4 Data collection

#### 2.4.1 General information collection

In this study, a structured questionnaire (Liu et al., 2009) was used to gather demographic information from participants, including gender, age, height, weight, body mass index (BMI), systolic blood pressure (SBP),diastolic blood pressure (DBP),and other relevant data. BMI was determined according to the formula: BMI = weight (kg)/[height(m)]^2^.

#### 2.4.2 Synchronous acquisition of PPW and ECG

**FIGURE 1:**
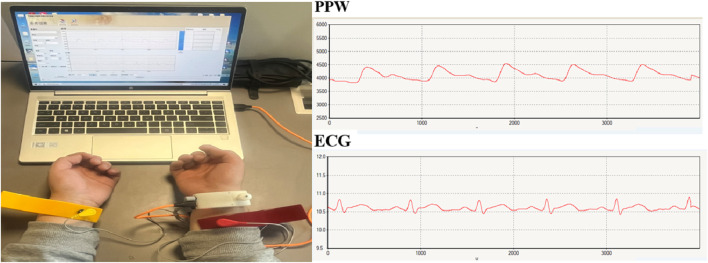
Synchronous acquisition of PPW and ECG signals.

Pulse diagnosis device (ZY-II type), equipped with pressure and electrocardiogram (ECG) sensors, was used to collect wrist pulse and limb-lead ECG signals. The device was jointly developed by Shanghai University of Traditional Chinese Medicine and East China University of Science and Technology, which acquisition terminal include pressure sensor and electrodes. The pressure sensor is placed at the strongest pulsation point of the wrist with a strap for PPW collection. The ECG acquisition terminal uses a standard limb-lead configuration for signal collection, with the red electrode positioned on the participant’s right upper limb, the yellow on the left upper limb, and the green on the right lower limb. Figure 1 presents an example.

Before data collection, all participants were instructed to rest for at least 3 min to ensure physiological stability. The data collection lasted for 60 s with a sampling frequency of 1,100 Hz. Optimal signals for subsequent feature extraction were collected when the signals from both channels with the software system exhibited stability and reached their maximum amplitude.

### 2.5 Data pre-processing

When acquiring PPW and ECG signals using hardware devices, high-frequency noise is initially removed through low-pass filtering. However, during the subsequent transmission process, these signals remain susceptible to various environmental interferences and power line interference, introducing both high-and low-frequency noise to varying degrees. This significantly impacts the subsequent signal analysis and processing tasks. Consequently, it is essential to apply digital filtering to the signals in order to eliminate baseline drift and ensure accurate analysis.

#### 2.5.1 Pre-processing for PPW

(i) Filtering of PPW Signals The PPW signal is a relatively weak physiological signal with its main frequency ranging from 0 to 20 Hz. The majority of its energy is concentrated within the 0–10 Hz range, and the dominant frequency energy is less than 3 Hz. In our experiment, the energy of the PPW signals is primarily distributed below 8 Hz. Therefore, a 3rd-order Butterworth low-pass filter with a cutoff frequency of 8 Hz was employed to filter the PPW signal. This filter effectively suppressed noise while preserving the integrity of the signal.
(ii) Removal of the Baseline Drift Following the low-pass filtering, while high-frequency noise in the PPW signal is effectively suppressed, the issue of baseline drift still lingers. The baseline drift observed in the PPW signal can be primarily attributed to two factors: interference from the human respiratory frequency and the sensitivity of the piezoresistive pulse sensor’s output waveform to pressure changes. The presence of baseline drift introduces a discernible fluctuation trend in the waveform, increasing the variability among pulse waveforms across different cycles. Such variability poses a significant challenge to subsequent feature extraction and signal processing analysis.

To address this issue, this study employs a cubic spline curve fitting method for baseline drift removal. Initially, the trough points in the PPW signal are identified. These trough points in the PPW signals are then utilized to perform cubic spline curve fitting, yielding the baseline drift curve. The advantage of cubic spline fitting lies in its ability to generate a smooth curve with gradual change. By subtracting this baseline drift curve from the original PPW signal, the PPW signal remains undistorted and is well-adjusted to the zero-line position. This provides a stable and accurate foundation for subsequent signal processing and analysis.

#### 2.5.2 Pre-processing for ECG

(i) Filtering of ECG Signals Upon examining the frequency spectrum of the collected ECG signals, it becomes evident that these signals are primarily affected by two types of interference: 50 Hz electromagnetic interference (EMI) originating from the electrical circuits and myoelectric interference with the frequency range of 20Hz–40 Hz. When determining the cutoff frequencies for the filters, it is necessary to balance between noise removal, smoothing of the ECG waveform, and preservation of the original signal morphology. These balances ensure that the subsequent identification of ECG feature points remains accurate and reliable.

To achieve this, a two-stage filtering method was designed in this study. In the first stage, a cutoff frequency of 40 Hz was set to effectively eliminate the 50 Hz power line noise. This step is essential for the accurate extraction of the R-wave feature points, which are critical for ECG analysis. Following, a cutoff frequency of 20Hz was applied in the second stage to further filter out any remaining noise above 20 Hz. By utilizing the signal from which noise above 20 Hz has been removed, other feature points were identified using the already detected R-waves as a reference. This two-stage approach ensures a clean and accurate ECG signal, facilitating the precise identification of all relevant feature points.

(ii) Removal of Baseline Drift Eliminating baseline drift in ECG signals is necessary for accurate analysis, and this can be achieved by adjusting the onset of the P-wave to the zero line. The key to accomplish this line in accurately identifying the onset of the P-wave in each ECG cycle. Once these points are determined, they can serve as the basis for fitting a baseline for the ECG signal.

In the specific implementation process, our study employed a low-pass filtering approach. Given that baseline drift typically occurs at relatively low frequencies, we applied a low-pass filter to the raw ECG signal. The cutoff frequency for this filter was carefully selected to be between 0.2 and 0.5 Hz, ensuring that the signal baseline could be effectively extracted. Subsequently, by subtracting this extracted baseline from the original signal, we obtained an ECG signal with the baseline drift removed, thereby enhancing the accuracy and reliability of subsequent analysis.

### 2.6 Feature extraction methods for PPW and ECG signals

#### 2.6.1 PPW feature extraction

**FIGURE 2:**
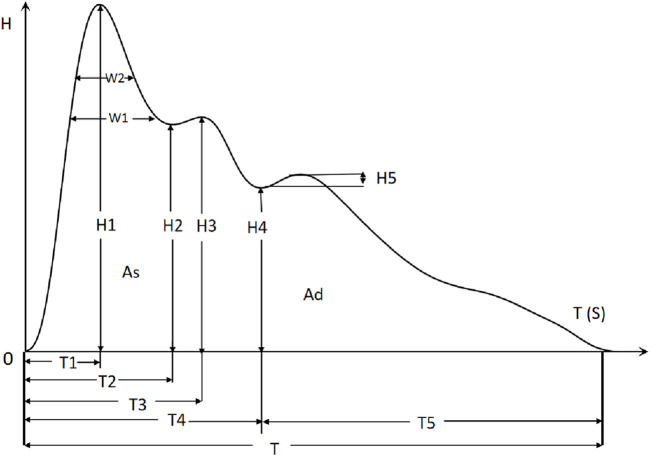
Time-domain analysis of PPW in typical circle.

In this study, we employ the time-domain analysis method to extract the peaks and troughs of PPW signal in typical cycle, thereby illustrating its amplitude (*H*_*1*_, *H*_*2*_, *H*_*3*_, *H*_*4*_, *H*_*5*_), duration (*T*_*1*_, *T*_*2*_, *T*_*3*_, *T*_*4*_, *T*_*5*_, T, *W*_*1*_, *W*_*2*_), and area feature (*A*_*s*_, *A*_*d*_), refer to Figure 2. Additionally, we calculated the ratios of these features (*H*_*2*_ */H*_*1*_, *H*_*3*_ */H*_*1*_, *H*_*4*_ */H*_*1*_, *H*_*5*_ */H*_*1*_, *T*_*1*_ */T*, *T*_*4*_ */T*, *T*_*1*_ */T*_*4*_, *T*_*5*_ */T*_*4*_, *W*_*1*_ */T*, *W*_*2*_ */T*, *A*_*s*_/*A*_*d*_) to gain further insights. Furthermore, pulse variation features such as P-rMSSD and P-SDNN were also calculated. The physiological significance of some statistically significant parameters is shown in Table 1 (Yan et al., 2021).

**TABLE 1:** Physiological significance of PPW features.

Features	Physiological significance
T	Reflects one cardiac cycle of the left ventricle
*T_1_*	Reflects left ventricular rapid ejection phase
*T_4_*	Reflects left ventricular systolic period
*T_5_*	Reflects left ventricular diastolic period
*W_1_*	The width of the upper 1/3 of the main wave indicates the duration of the artery’s high-pressure level
*W_2_*	The width of the upper 1/5 of the main wave indicates the duration of the artery’s high-pressure level
*H_2_/H_1_*	Reflects arterial elasticity and peripheral vascular resistance
*H_3_/H_1_*	Reflects arterial elasticity and peripheral vascular resistance
*H_5_/H_1_*	Reflects aortic elasticity and aortic valve function
*T_1_/T*	Reflects cardiac ejection function
*T_4_/T*	Reflects relative duration of the systole
*W_1_/T*	Reflects the duration of the artery’s high-pressure level
*W_2_/T*	Reflects the duration of the artery’s high-pressure level
P-rMSSD	Reflects root mean square of the difference between adjacent pulsation periods
P-SDNN	Reflects the standard deviation of the adjacent pulsation period

#### 2.6.2 ECG feature extraction

**FIGURE 3:**
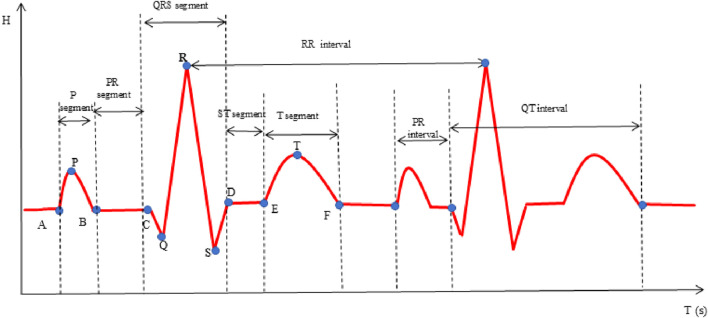
Time-domain analysis of ECG.

In this investigation, the time-domain method was used to extract ECG-specific points, as illustrated in Figure 3 (Xu et al., 2017). Subsequently, a comprehensive of features was calculated, including the P, Q, R, S, T waves, along with various segments and intervals: P segment, QRS segment, T segment, PR segment, ST segment, PR interval, QT interval, RR interval and heart rate (HR). Additionally, heart rate variability (HRV) features SDNN, RMSSD and mean RR were calculated. The physiological significance of some statistically significant parameters is shown in Table 2.

**TABLE 2:** Physiological significance of ECG features.

Features	Physiological significance
QRS segment	Reflects the completion time of ventricular depolarization
PR interval	Reflects ventricular activation time
ST segment	Reflects the time from ventricular depolarization to the beginning of repolarization
QT interval	Reflects the total time of ventricular depolarization and repolarization
*HR*	Reflects the number of beats per minute at rest
RR interval	Reflects the time between consecutive heartbeats, starting from the activation of the sinoatrial node
*SDNN*	Reflects overall heart rate variability, indicating the autonomic nervous system’s regulation
RMSSD	Indicates short-term heart rate variability, primarily reflecting parasympathetic activity
mean RR	Reflects the mean of sinus RR intervals

### 2.7 Statistical analysis

Statistical analysis was conducted using SPSS Statistics 25.0 (IBM, Armonk, NY, United States) to compare differences in pulse and ECG features among the four groups. For continuous variables, if the data followed a normal distribution, analysis of variance (ANOVA) was utilized, with results expressed as mean and standard deviation (denoted as x¯±SD); if the assumption of normal distribution was not met, the non-parametric Mann-Whitney U test was used, with outcomes represented by the median and quartiles, (denoted as M (QR1-QR3)). For categorical data, the Chi-square test was employed, with results expressed in terms of frequencies and percentages (denoted as n (%)). A significance level of P < 0.05 was used to indicate statistical significance.

### 2.8 Model establishment and evaluation methods

#### 2.8.1 Model establishment

In this study, three distinct machine learning algorithms were employed to develop models for disease identification. These algorithms include Random Forest (RF), Support Vector Machine (SVM), and Bagged Decision Trees (BDT). RF is an ensemble learning method that combines predictions from multiple decision trees, determining the final result by selecting the most frequent outcome among these trees (Schwarz et al., 2010). This approach leverages the strength of multiple weak learners to improve predictive accuracy.

BDT, on the other hand, employs the technique of Bagging or Bootstrap Aggregating, which involves generating multiple versions of a decision tree predictor by resampling the training data and then aggregating their predictions to obtain a final result (Chen and Guestrin, 2016). Bagging helps to reduce overfitting and improve the stability of the mode.

SVM is a powerful supervised learning algorithm use for classification and regression tasks. It seeks to find an optimal hyperplane within the feature space, maximizing the margin between distinct class (Boser et al., 1992). SVM can handle both linear and non-linear data through the use of kernel functions, which transform the input data into a higher-dimensional space where a linear separation is possible. Each of these models possesses unique strengths and has found widespread application in predictive analytics.

#### 2.8.2 Model evaluation methods

A confusion matrix was used to summarize performance of a classification model. This matrix compares the actual class of an instance with the class predicted by the model. The matrix typically has two dimensions: the classes and the predicted classes. A typical structure of the confusion matrix for a binary classification problem is presented in Table 3.

**TABLE 3:** Confusion matrix.

Predicted classes	Actual classes	
	Positive	Negative
Positive	TP	FP
Negative	FN	TN

Accuracy: The ratio of correctly predicted instances (true positives and true negatives) to the total number of instances (predictions).


$$\text{Accuracy}=\frac{TP+TN}{TP+FN+FP+TN}$$ (1)

Using the confusion matrix, calculated accuracy, precision, recall and F1-score employing Formula 1 through Formula 4, respectively. These evaluation metrics are equally applicable to multi-classification problems.

Precision: The ratio of correctly predicted positive instances to the total number of instances predicted as positive.

$$\text{Precision}=\frac{TP}{TP+FP}$$ (2)

Recall: The ratio of correctly predicted positive to the total number of actual positive.

$$\text{Recall}=\frac{TP}{TP+FN}$$ (3)

F1-Score: The harmonic means of precision and recall.

$$F1-\text{score}=\frac{2\times \text{Precision}\times \text{Recall}}{\text{Precision}+\text{Recall}}$$ (4)

## 3 Results

### 3.1 Sample size calculation

To ensure sufficient statistical power for our study design, we conducted sample size calculations using G*Power 3.1.9.7 software t. Based on Cohen’s (Cohen, 2013) recommendations, we adopted a medium effect size (w = 0.30), a significance level (α) of 0.05, and a power (1 − β) of 0.80. The degrees of freedom were calculated as (4–1) × (27–1) = 78. Based on these parameters, our sample size calculation indicated that a minimum of 405 participants were required. Ultimately, we recruited total of 463 participants, thus meeting the sample size requirement for robust statistical analysis.

### 3.2 Demographic comparison among groups

The demographic characteristic of the study groups is presented in Table 4. Upon statistical analysis, no significant differences in sex distribution were observed among the groups (P > 0.05). However, a notable age discrepancy was observed, whereby individuals in Group 2, Group 3, and Group 4 were exhibited significantly higher mean age than those in Group 1 (P < 0.05). Additionally, the BMI values in Group 3 and Group 4 were significantly higher compared to both Group 1 and Group 2 (P < 0.05). This indicates a higher prevalence of overweight or obesity in.

**TABLE 4:** Comparison of demographic data among groups [*n* (*%*),*‾x±SD*].

Groups	n	Sex		Age	BMI	SBP (mmHg)	DBP (mmHg)
		Male	Female				
Group 1	152	96 (63.2%)	56 (36.8%)	44.85 ± 21.86	23.13 ± 2.95	125.59 ± 16.39	74.82 ± 8.49
Group 2	75	38 (50.7%)	37 (49.3%)	65.25 ± 11.35*	23.29 ± 3.72	127.96 ± 16.88	76.84 ± 9.24
Group 3	134	75 (56.0%)	59 (44.0%)	68.95 ± 8.39*	24.22 ± 3.35*#	134.73 ± 14.71*#	77.95 ± 10.29*
Group 4	102	58 (56.8%)	44 (43.2%)	68.74 ± 7.85*	24.47 ± 2.99*#	137.75 ± 14.78*#	79.48 ± 9.25*
Statistical value	--	x2 = 3.60		F = 87.12	F = 5.07	F = 15.79	F = 5.64
*P* value	--	0.31		<0.001	<0.001	<0.001	<0.001

Note: *, compared with Group 1, P < 0.05; #, compared with Group 2, P < 0.05.

Two groups relative to the others. Additionally, significant higher levels of SBP and DBP were observed in Group 3 and Group 4 when compared to Group 1 (P < 0.05). These findings suggest potential variations in cardiovascular health status among the study groups.

### 3.3 Comparison of PPW and ECG features among groups

The analytic results of PPW and ECG features across the four groups is presented in Table 5 and 6, respectively. Table 5 revealed that compared to Group 1 with healthy individuals, all other groups demonstrated significant increases in various pulse features, including *H*_*2*_ */H*_*1*_, *H*_*3*_ */H*_*1*_, *T*, *T*_*1*_, *T*_*4*_, and *T*_*5*_, while *H*_*5*_ */H*_*1*_ was notably lower. These findings suggest altered hemodynamics in the groups with CHD and its comorbidities. Specifically, the increased *H*_*2*_ */H*_*1*_ and *H*_*3*_ */H*_*1*_ ratios indicated reduced arterial elasticity and elevated peripheral vascular resistance, whereas the decrease in *H*_*5*_ */H*_*1*_ may suggest impaired aortic elasticity and aortic valve function.

**TABLE 5:** Comparison of PPW features among groups [*M*(*QR*_*1*_ *-QR*_*3*_)].

Pulse features	Group 1 (n = 152)	Group 2 (n = 75)	Group 3 (n = 134)	Group 4 (n = 102)	*Z*	*P*
*H_2_/H_1_*	0.85 (0.56,0.95)	0.94 (0.86,0.97)*	0.92 (0.84,0.96)*	0.93 (0.89,0.97)*	31.97	<0.001
*H_3_/H_1_*	0.72 (0.46,0.86)	0.83 (0.73,0.89)*	0.81 (0.72,0.86)*	0.83 (0.74,0.88)*▲	28.77	<0.001
*H_5_/H_1_*	0.38 (0.30,0.43)	0.3 (0.25,0.36)*	0.34 (0.27,0.40)*	0.32 (0.25,0.37)*▲	35.02	<0.001
*T*	0.79 (0.72,0.88)	0.86 (0.78,0.93)*	0.9 (0.80,1.00)*#	0.85 (0.74,0.92)*#▲	34.67	<0.001
*T_1_*	0.13 (0.12,0.14)	0.15 (0.14,0.16)*	0.14 (0.13,0.16)*	0.14 (0.13,0.16)*#	33.18	<0.001
*T_4_*	0.32 (0.30,0.35)	0.35 (0.33,0.37)*	0.36 (0.33,0.38)*	0.34 (0.32,0.36)*#	45.63	<0.001
*T_5_*	0.41 (0.39,0.45)	0.46 (0.43,0.48)*	0.46 (0.42,0.47)*	0.44 (0.41,0.46)*#▲	63.36	<0.001
*T_1_/T*	0.16 (0.14,0.19)	0.18 (0.15,0.20)*	0.16 (0.14,0.18)#	0.17 (0.15,0.19)*▲	12.11	<0.001
*W_1_*	0.16 (0.12,0.20)	0.19 (0.17,0.22)*	0.2 (0.17,0.23)*#	0.19 (0.17,0.22)*	58.79	<0.001
*W_2_*	0.11 (0.08,0.15)	0.14 (0.12,0.17)*	0.15 (0.12,0.19)*#	0.15 (0.12,0.18)*	57.78	<0.001
*W_1_/T*	0.21 (0.15,0.24)	0.24 (0.19,0.25)*	0.23 (0.20,0.25)*	0.23 (0.20,0.25)*	24.22	<0.001
*W_2_/T*	0.15 (0.10,0.18)	0.17 (0.14,0.20)*	0.17 (0.13,0.20)*	0.18 (0.15,0.20)*#▲	33.03	<0.001
*P*-rMSSD	36.73 (24.67,60.73)	20.68 (13.14,80.10)	22.25 (13.52,40.81)*	17.14 (10.67,40.62)	14.67	<0.001
*P* -SDNN	36.73 (24.67,60.73)	23.74 (15.50,74.21)	23.48 (15.92,43.79)*	21.46 (15.12,36.21)*#	23.81	<0.001

Note: *, compared with Group 1, P < 0.05; #, compared with Group 2, P < 0.05; ▲, compared with Group 3, P < 0.05.

**TABLE 6:** Comparison of ECG features among groups [*M*(*QR*_*1*_ *-QR*_*3*_)].

ECG Features	Group 1 (n = 152)	Group 2 (n = 75)	Group 3 (n = 134)	Group 4 (n = 102)	Statistical value	*P*
QRS segment	0.072 (0.057,0.081)	0.078 (0.071,0.095)*	0.077 (0.063,0.084)	0.075 (0.068,0.093)*	Z = 16.94	<0.001
ST interval	0.085 (0.077,0.093)	0.092 (0.076,0.104)*	0.095 (0.085,0.105)*	0.095 (0.078,0.108)*	Z = 14.89	<0.001
PR interval	0.137 (0.115,0.163)	0.165 (0.143,0.185)*	0.163 (0.141,0.191)*	0.16 (0.135,0.188)*	Z = 9.77	<0.001
QT interval	0.366 (0.314,0.402)	0.40 (0.375,0.434)*	0.404 (0.368,0.431)*	0.387 (0.354,0.441)*	Z = 8.11	<0.001
HR	76.22 ± 11.72	74.91 ± 11.06	73.66 ± 17.74*	78.24 ± 15.13▲	F = 2.097	0.009
mean RR	781.51 (723.404,858.20)	788.85 (732.68,892.04)	833.25 (756.87,931.19)	782.9 (709.85,867.29)*▲	Z = 11.76	0.002

Note: *, compared with Group 1, P < 0.05; #, compared with Group 2, P < 0.05; ▲, compared with Group 3, P < 0.05.

When Compared to Group 1, Group 2 exhibited a higher *T*_*1*_ */T* ratio, reflecting an elevated cardiac ejection function in CHD patients. This increase is likely a compensatory mechanism, suggesting that the heart is working hard to maintain normal blood supply. Additionally, Group 3, when compared to Group 1, exhibited lower *P*-rMSSD and *P*-SDNN values, signifying reduced pulse rate variability. This reduction in pulse rate variability mirrors changes in HRV to some extent and suggest a decline in autonomic nervous function of the heart.

Furthermore, the P-SDNN value in Group 4 was even lower than that in Group 3, further emphasizing the progressive decline in autonomic function with the addition of diabetes as a comorbidity. When compared to Group 2,Group 3 showed higher values for *T*, *W*_*1*_, and *W*_*2*_, suggesting a decreased in HR and an increased in cardiac afterload in CHD patients with hypertension. These changes are associate with an elevated risk of adverse cardiovascular events.

Lastly, Group4 exhibited a decreased T_1_/T ratio compared to Group 2, suggesting impaired cardiac function in the CHD patients with both hypertension and diabetes. These findings underscore the complex interplay between CHD, its comorbidities, and the resulting changes in cardiac function and hemodynamics.

In the analysis of ECG features presented in Table 6, all groups, with the exception of Group1 demonstrated elevated ST, PR, and QT intervals. These findings are indicative of potential conduction abnormalities in the groups with CHD and its comorbidities. Compared to Group 1, Group 2 and Group 4 exhibited increased QRS segments, suggesting prolonged ventricular depolarization. In contrast, Group 3 exhibited a significantly reduced heart rate (HR), which is likely attributable to autonomic dysfunction. Group 4 also displayed increased QRS segments and mean RR intervals. With a higher HR and lower mean RR intervals than Group 3, reflecting the additional impact of diabetes on cardiac electrical activity and heart rate regulation. These findings highlight distinct ECG changes associated with varying cardiovascular and metabolic conditions.

### 3.4 Establishment and comparison of models

#### 3.4.1 Using SMOTE to balance the dataset for classification

In this study, we have four distinct groups, comprising a total of 463 samples for modeling. However, the sample distribution across the four groups is unbalanced. It is well-recognized fact in the research community that achieving a balanced sample size among different groups is crucial for improving model generalization and reducing the bias induced by class imbalance. To address the imbalance across the four sample groups in the dataset,we employed the Synthetic Minority Over-sampling Technique (SMOTE). This method effectively balances the dataset by generating synthetic samples of the minority class (Dablain et al., 2023). In implementing SMOTE, we focused on augmenting only the minority class data. We adjusted the sampling ratio to guarantee that the augmented minority class matched the quantity to the majority class (n = 152). As for the parameter K, which represents the number of nearest neighbors considered in the synthesis process, we assigned a value of 5. By applying SMOTE, we effectively enriched our dataset, thereby establishing a more balanced data foundation for subsequent classification tasks.

**FIGURE 4:**
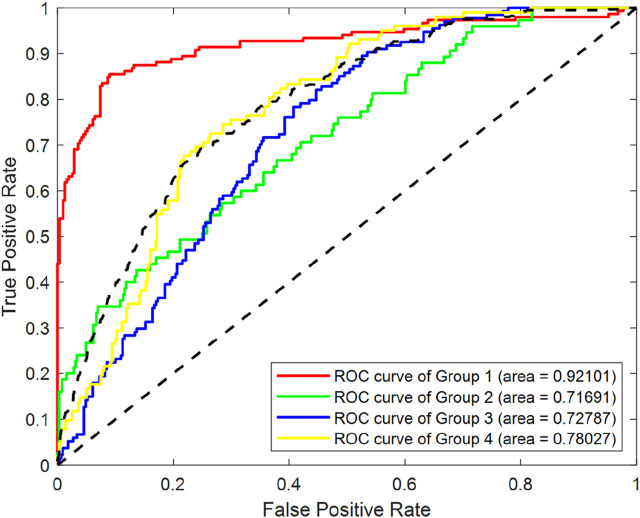
ROC curves of RF classification before SMOTE processing.

To systematically evaluate the effect of SMOTE processing, we conducted a comparative analysis using a RF classifier, utilizing the entirety of the original dataset. As depicted in Figure 4, which displays the Receiver Operating Characteristic (ROC)curves of Random Forest (RF) classification before SMOTE was applied, we can observe the classification performance of the original imbalanced dataset. The Area Under the ROC Curve (AUC) values for Group1, Group2, Group3 and Group4 were 0.92101, 0.71691, 0.72787 and 0.78027 respectively.

**FIGURE 5:**
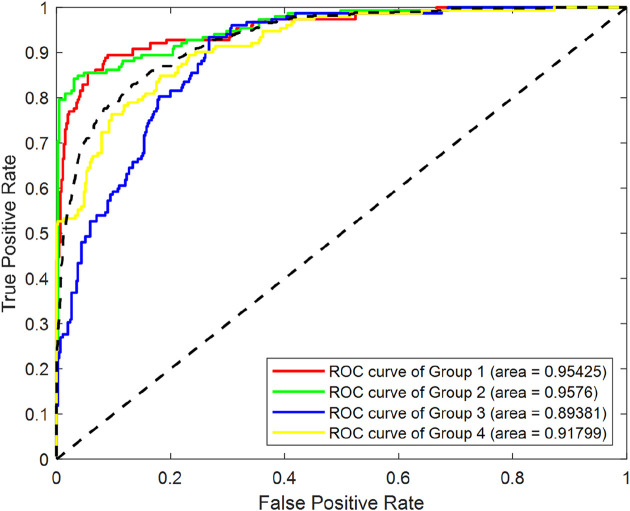
ROC curves of RF classification after SMOTE processing.

Figure 5 illustrates the ROC curves of RF classification after SMOTE processing. The AUC values for Group1, Group2, Group3 and Group4 were found to be 0.95425, 0.9576, 0.89381 and 0.91799 respectively. This represents an improvement of 0.033, 0.241, 0.166, and 0.138 for the four groups, respectively.

In summary, after achieving data balancing through SMOTE, the classification performance of the model has been enhanced, demonstrating the effectiveness of this technique in addressing class imbalance issues.

#### 3.4.2 Establishment and comparison of the RF models based on different datasets

After balancing sample size of different groups, we proceeded with modeling. The RF algorithm was select for modeling due to its robustness and strong performance in handling complex datasets. To further minimize the risk of overfitting and to validate the robustness of our models, we utilized a 5-fold cross-validation approach. In this method, the dataset is divided into five equal subsets, with four parts being used for training and the remaining one part for testing. This is process is repeated five times, with a different subset serving as the test set in each iteration.

To compare the impact of different physiological signals on prediction models for CHD and its associated comorbidities, we designed and established multiple models using various datasets. These datasets were derived from simultaneously acquired PPW and ECG signals. They encompassed PPW features, ECG features, as well as a combination of both. The first RF model, designated as Model 1, was constructed using a dataset that included demographic data alongside ECG features. The second model, named Model 2, incorporated demographic data and PPW features. Lastly, the third RF model, referred to as Model 3, was established based on the comprehensive original dataset, which included both PPW and ECG features along with demographic data.

**FIGURE 6:**
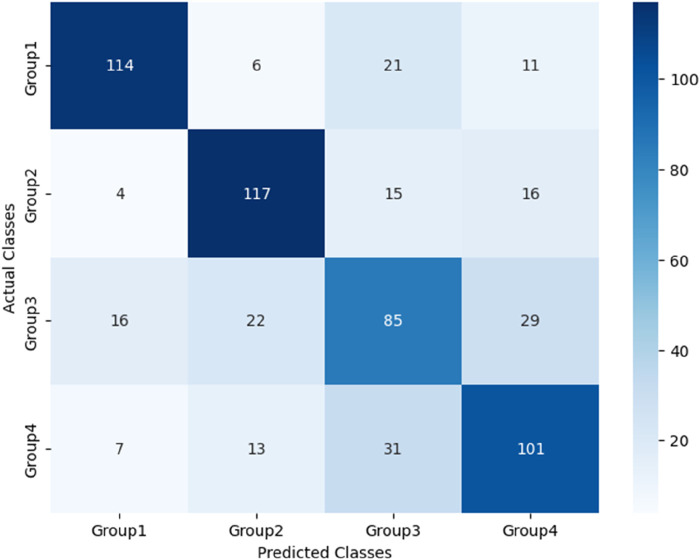
Confusion matrix for Model 1 based on PPW features.

**FIGURE 7:**
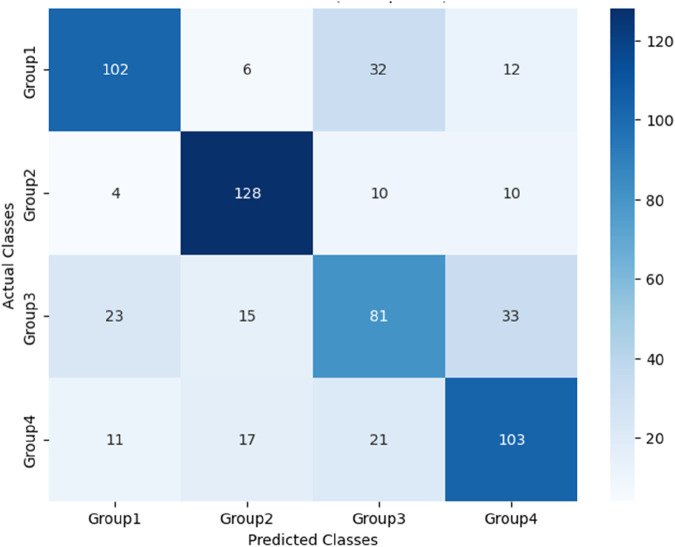
Confusion matrix for Model 2 based on ECG features.

**FIGURE 8:**
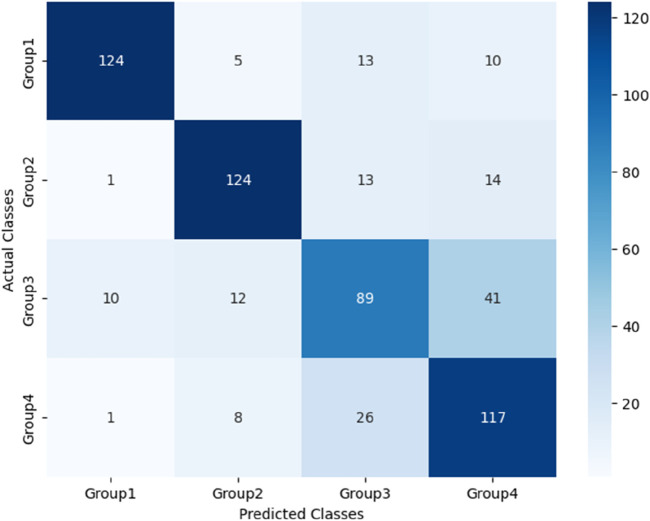
Confusion matrix for Model 3 based on PPW and ECG features.

**FIGURE 9:**
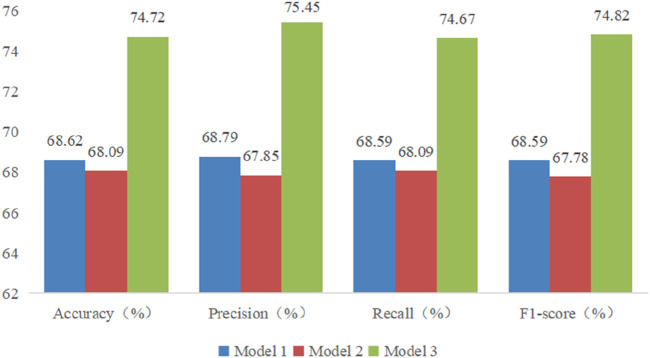
Comparison of models based on differrent datasets.

The performance of these RF models was assessed using accuracy, precision, recall, and F1-score metrics, all of which were calculated based on confusion matrices (as depicted Figures 6–8) following formulas outlined in Section 2.8.2. Our study found that Model 3, which was built on the complete original dataset, achieved the best performance. Specifically, it achieved an accuracy of 74.72, a precision of 75.45%, a recall of 74.67%, and a F1-score of 74.84%. These metrics represent substantial improvements compared to the other models: When compared to Model 1, the improvements were 6.10% in accuracy, 6.66% in precision, 6.08% in recall, and 6.23% in F1-score. Furthermore, when compared to Model 2, the enhancements were even more pronounced, with increases of 6.63% in accuracy, 7.6% in precision, 6.58% in recall, and 7.04% in F1-score. A detailed summary of the comparison of RF models based on different datasets was presented in Figure 9.

#### 3.4.3 Establishment and comparison of models utilizing different algorithms

##### 3.4.3.1 Hyperparameter optimization of different models

In this section, we present the establishment and comparative analysis of models constructed using various algorithms. The objective was to assess the performance of different computational methods in predicting the CHD and its associated comorbidities.

In addition to the baseline random forest model (referred to as Model 3 in Section 3.4.2), which incorporating PPW, ECG, and demographic features, we developed the modes using support vector machine (SVM), bagged decision tree (BDT) and A feature-selected RF model (FS-RF) using the same dataset.

In this study, we employed the Grid Search method to optimize the hyperparameters of the RF, SVM, and BDT models. The specific configurations are outlined below: For the RF and BDT models, the hyperparameters that we optimized include the number of trees (with a search range set from 50 to 200) and the minimum number of samples required at a leaf node (with a search range set from 1 to 3). By fine-tuning these parameters, we aimed to find an optimal balance that ensures the model sufficiently learns the data while avoiding overfitting. For the SVM model, the hyperparameters that we optimized are the penalty coefficient C (with a search range of 10^–8^ to 10^8^) and the kernel coefficient gamma (with a search range of 10^–8^ to 10^8^). Through Grid Search, we were able to identify the hyperparameters that yield the best performance of the model on the validation set.

##### 3.4.3.2 RF model with feature selection

**FIGURE 10:**
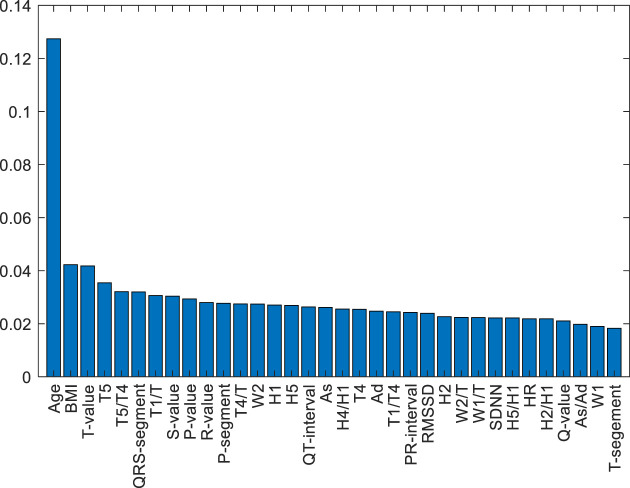
SF-RF model ranked the features based on their importance scores.

**FIGURE 11:**
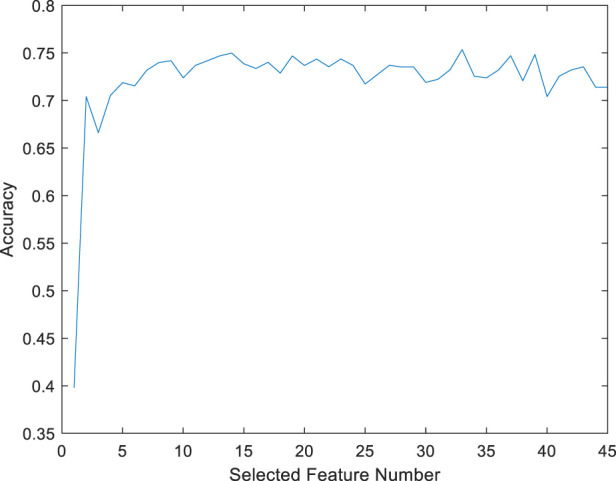
RS-RF model accuracy based on different selected features.

A feature-selected RF model (FS-RF) was constructed based on importance scores generated by the RF algorithm. For the FS-RF model, we adopted a systematic feature selection approach combining feature importance ranking and Sequential Forward Selection (SFS). First, all features were ranked based on their importance scores calculated by the RF algorithm, as shown in Figure 10. Subsequently, SFS was applied to iteratively incorporate features while evaluating model performance using 5-fold cross-validation accuracy. The analysis revealed that the model achieved peak accuracy when the top 34 most important features were included, as demonstrated in Figure 11. Further addition of features led to a decline in validation accuracy, indicating the onset of overfitting. This optimal feature subset achieved a balance between predictive performance and model simplicity, making it an ideal choice for our prediction task.

##### 3.4.3.3 Performance comparison of different models

To begin with,the dataset underwent appropriate preprocessing. The SMOTE was employed to address class imbalance. Subsequently, the dataset was partitioned into training and testing sets. All models underwent optimized through 5-fold stratified cross-validation.

**FIGURE 12:**
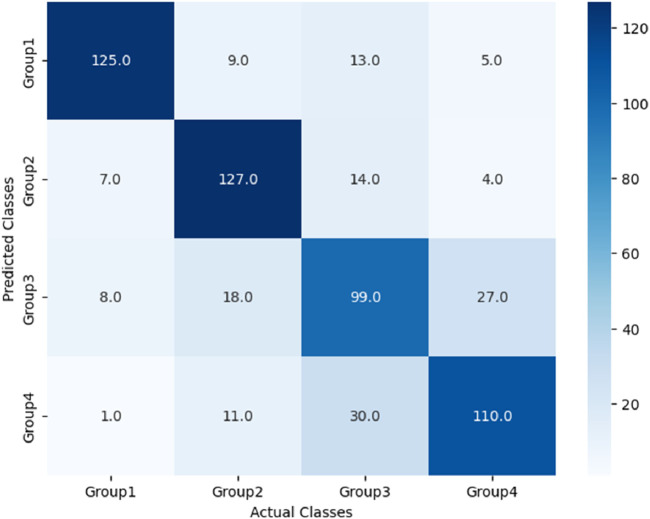
Confusion matrix of FS-RF model.

**FIGURE 13:**
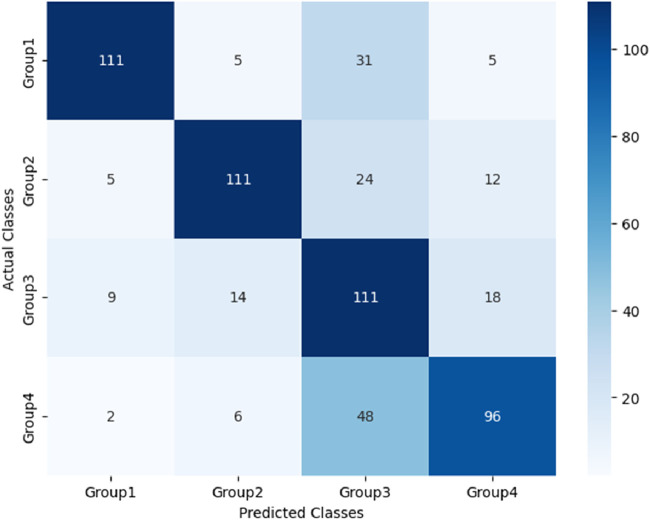
Confusion matrix of SVM model.

**FIGURE 14:**
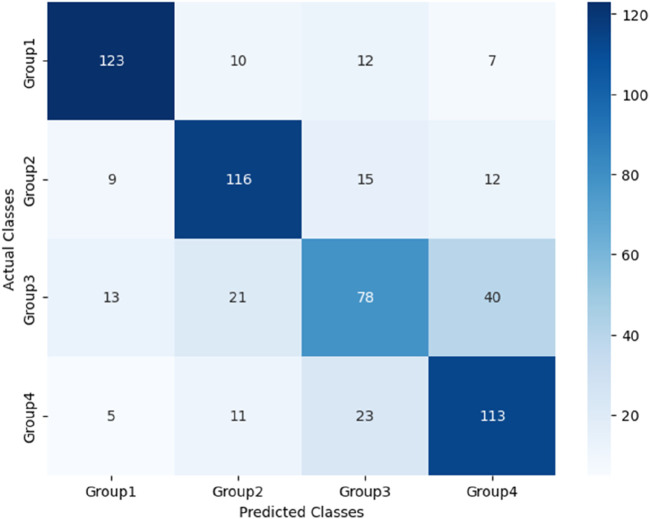
Confusion matrix of BDT model.

After the models were established, a comparison was conducted to evaluate their predictive performance. Confuse matrices and their associated performance metrics were employed to quantify and compare the models’ effectiveness. The prediction results of different models were visualized using confusion matrices, as depicted in Figures 8, 12–14(which presents the results for the RF model without feature selection, the FS-RF model, the SVM model and BDT model). Based on these confusion matrices, we computed several key metrics, including accuracy, average precision, average recall, and average F1-scores, to provide a comprehensive assessment of model performance.

As demonstrated in Table 7, our study finding revealed that the FS-RF model, after undergoing feature selection, achieved an accuracy of 76.32%, an average precision of 75.82%, an average recall of 76.11%, and average F1-scores of 75.88%. When compared to the RF model without feature selection, the FS-RF model demonstrated improvement 1.60% in accuracy, 0.37% in average precision, 1.44% in average recall, and 1.06% in average F1 score.

**TABLE 7:** Performance comparison of four models.

Classifier	Group	Precision (%)	Recall (%)	F1-score (%)	Average Precision (%)	Average Recall (%)	Average F1-score (%)	Accuracy (%)
FS-RF	Group1	82.24	88.65	85.29	75.82	76.11	75.88	76.32
	Group2	83.55	76.97	80.12				
	Group3	65.13	63.46	64.28				
	Group4	72.37	75.34	73.81				
RF	Group1	91.18	81.58	86.33	75.45	74.67	74.82	74.72
	Group2	83.22	81.58	82.34				
	Group3	63.12	58.55	60.74				
	Group4	64.29	76.97	69.86				
SVM	Group1	73.03	87.40	79.57	70.56	73.54	71.32	70.56
	Group2	73.03	81.62	77.34				
	Group3	73.03	51.87	60.59				
	Group4	63.16	73.28	67.78				
BDT	Group1	80.92	82.00	81.32	70.72	70.52	70.34	70.72
	Group2	76.32	73.42	74.38				
	Group3	51.32	60.94	55.83				
	Group4	74.34	65.70	69.83				

Moreover, the FS-RF model significantly outperformed SVM and BDT models across all performance metrics. In terms of accuracy, the FS-RF model achieved a 5.76% and 5.60% improvement compared to SVM and BDT models, respectively. Similarly, it exhibited a 5.26% and 5.10% increase in the average precision, a 2.57% and 5.59%, improvement in average recall, and a 4.56% and 5.54% rise in average F1-score when compared to SVM and BDT models, respectively.

To statistically validate these performance differences, paired t-tests were conducted on the cross-validation accuracies. The results revealed statistically significant performance improvements. Specifically, the FS-RF model outperformed the RF model (*p* = 3.56 × 10^−6^), the SVM model (*p* = 3.31 × 10^−7^), and the BDT model (*p* = 5.17 × 10^−6^). These findings clearly demonstrate that the feature selection strategy significantly enhanced RF model’s predictive performance.

These comparative results highlight the significant performance advantages of the RF algorithm in classification tasks. Particularly, when optimized with appropriate feature selection techniques, the performance of the FS-RF algorithm achieves even better performance. This emphasizes the importance of feature selection in improving the predictive capabilities of machine learning models for classification.

#### 3.4.4 External validation of the FS-RF model

To evaluate the generalizability of the FS-RF model, an independent external validation cohort was collected from Jiading District Central Hospital, Shanghai. This cohort consisted of 285 participants, comprising 58 healthy controls, 99 individuals diagnosed with CHD, 106 individuals with both CHD and hypertension, and 22 individuals with a combination of CHD, hypertension and diabetes.

In the external validation set, the FS-RF model achieved an overall accuracy of 81.27%. The class-specific accuracies were as follows: 87.93% for healthy controls, 97.98% for individuals with CHD alone, 66.04% for individuals with both CHD and hypertension, and 60.0% for individuals with CHD, hypertension and diabetes.

## 4 Discussion

Hypertension and diabetes are significant risk factors for CHD. These conditions, along with CHD, interact in a complex and interdependent manner, with each amplifying the adverse effects of the others. Prolonged hypertension can lead to the narrowing or blockage of coronary arteries, subsequently causing ischemia and hypoxia in myocardial cells, thereby accelerating the process of atherosclerosis (Poznyak et al., 2022). Moreover, diabetes contributes to vascular endothelial damage and promotes inflammation, furthering arteriosclerosis by impairing the coagulation mechanism (Dubsky et al., 2023; Yang et al., 2024). These conditions form a detrimental cycle that exacerbates atherosclerosis and ultimately leading to advanced coronary artery disease. Early identification of CHD and its comorbidities is important.

This study utilizes a wearable multi-source sensor pulse diagnostic device to collect pulse PPW and ECG signals from patients with CHD and those with comorbid hypertension or diabetes, followed by the extraction of signal features. Based on these features and individual information (such as age, BMI, and other cardiovascular risk factors), we employ multiple machine learning algorithms to construct predictive models for CHD and its comorbidities. The performance of different models is then compared. The study aims to provide a non-invasive, convenient, and real-time monitoring method for the early clinical diagnosis of CHD and its comorbidities.

The results of this study show that there are differences in PPW and ECG characteristics among different groups. These variations in physiological signals reflect pathological changes associated with CHD and its comorbidities. (1) Regarding the correlation between PPW and vascular function: For example, compared with healthy individuals, CHD patients and those with comorbidities showed increased pulse wave characteristics *H*_*2*_ */H*_*1*_ and *H*_*3*_ */H*_*1*_, suggesting reduced arterial elasticity and elevated peripheral vascular resistance. The decreased *H*_*5*_ */H*_*1*_ may indicate impaired aortic elasticity and aortic valve dysfunction in CHD and its comorbidities. Furthermore, compared with the CHD group, the CHD with hypertension group exhibited increased *W*_*1*_ and *W*_*2*_ in PPW features, indicating elevated cardiac afterload, which suggests that long-term poorly controlled blood pressure may lead to changes in left ventricular systolic function. (2) Regarding ECG changes and myocardial electrophysiological alterations: For example, compared with healthy individuals, CHD patients and those with comorbidities exhibited abnormal ECG features: prolonged ST segment suggesting myocardial ischemic injury, prolonged PR interval reflecting atrioventricular conduction dysfunction, and prolonged QT interval potentially associated with abnormal ventricular repolarization. Additionally, the CHD with hypertension and diabetes group showed increased mean RR interval. Heart rate variability analysis indicated that this change was related to cardiac autonomic neuropathy, possibly due to decreased sympathetic activity or increased parasympathetic tone, leading to bradycardia. This finding is consistent with previous research (Duan et al., 2023). This study confirms that synchronously acquired multimodal PPW and ECG data can complement each other’s advantages, providing multidimensional diagnostic information for early identification of CHD and its comorbidities, as well as references for understanding disease progression.

In terms of model construction, this study explored two key aspects: physiological signal fusion and algorithm optimization, with the following findings:

(1) The enhancement effect of multimodal physiological signal fusion on model identification. Through comparative analysis of modeling performance between models based on single signal source and multimodal signals, this study confirmed the clinical value of multidimensional feature collaborative diagnosis. The identification models for CHD and its comorbidities established based on synchronously acquired PPW and ECG information demonstrated superior performance to models using single signal modality. Compared with models built solely on ECG signals, the multimodal models showed improvements of 6.10% in accuracy, 6.66% in precision, 6.08% in recall, and 6.23% in F1-score. When compared with models relying solely on PPW signals, these four metrics improved by 6.63%, 10.90%, 6.58%, and 7.04% respectively. These results demonstrate that multimodal signal fusion enables more comprehensive evaluation of patients’ physiological status, significantly enhancing the diagnostic and predictive capabilities of the models. The performance improvements were consistent across all evaluation metrics, particularly showing notable enhancement in precision (10.90% increase compared to PPW-only models), suggesting that multimodal integration effectively reduces false positive rates in disease identification.
(2) Impact of machine learning algorithm optimization on model performance. This study further compared the performance of four machine learning algorithms (FS-RF, RF, SVM, BDT) on the multimodal PPW and ECG dataset, highlighting the importance of feature engineering and algorithm compatibility. The results demonstrated that the FS-RF model achieved the best performance, with accuracy, average precision, average recall, and average F1-score of 76.32%, 75.82%, 76.11%, and 75.88%, respectively. Compared to the standard RF model without feature selection, these metrics improved by 1.60%, 0.37%, 1.44%, and 1.06%, respectively. In contrast, SVM and BDT models exhibited relatively inferior performance.

This study demonstrated the advantages of the RF algorithm in handling high-dimensional data, as its built-in feature importance evaluation mechanism provided an objective basis for feature selection (Yaqoob et al., 2025). Specifically, the FS-RF model, constructed using 34 key features selected through this approach, achieved optimal performance. These findings suggest that proper feature selection combined with algorithm optimization significantly enhances model efficacy in CHD and comorbidity identification.

The top 34 features selected by the FS-RF model based on feature importance ranking show high correlation with cardiovascular disease mechanisms and possess distinct clinical significance. For example, Age and BMI, as traditional cardiovascular risk factors, ranked as the top two most important features. The PPW time-domain feature *H4/H1* negatively correlates with vascular compliance, reflecting arterial stiffness; *W1/T* and *W2/T* are associated with arterial pressure waveform variations, indicating peripheral resistance changes (Zhang et al., 2021). The ECG heart rate variability index SDNN reduction reflects autonomic nervous dysfunction, consistent with pathological characteristics of coronary heart disease complicated by hypertension or diabetes (Fitzpatrick et al., 2018). *P-wave* amplitude (*P-value*) and duration (*P-segment*) can assess atrial structure and functional status.

These features are directly linked to pathological mechanisms, not only improving classification accuracy but also enhancing the model’s clinical interpretability, highlighting the crucial role of feature engineering in model optimization and clinical translation.

Through the feature selection process, we effectively eliminated redundant or irrelevant features, simplified the model structure, and improved computational efficiency and interpretability (Li and Mu, 2024). The independent external validation cohort evaluation in this study demonstrated that the FS-RF model achieved an overall accuracy of 81.27%, indicating good generalization capability. While the model performed excellently in distinguishing healthy individuals from pure CHD patients, its performance declined slightly in identifying complex cases involving CHD with comorbid hypertension and diabetes. This may be attributed to increased clinical heterogeneity, feature overlap, and sample size imbalance (Abdalrada et al., 2022).

Although the study achieved certain results, several limitations require further optimization in subsequent research. First, the insufficient total sample size and imbalanced inter-group distribution affected the model’s stability and generalization ability. Second, the limitation in synchronous PPG and ECG acquisition duration. To balance experimental rigor with clinical feasibility, this study adopted a 60-s synchronous acquisition protocol for PPG and ECG signals. However, this duration may not fully capture patients’ pathological characteristics. The 60-s selection was based on considerations that the study population consisted of hospitalized patients with generally complex health conditions, where prolonged data collection might cause discomfort, reduce compliance, and increase the probability of motion artifacts.

Future research will focus on the following improvements: 1) multi-center data collection to expand sample size for building more robust models; 2) optimize signal acquisition protocol design through dynamic adjustment of collection duration to enhance transient physiological event detection while maintaining patient comfort; and 3) enhancing signal processing techniques to effectively eliminate complex environmental noise interference and motion artifacts in clinical settings.

## 5 Conclusion

This study aims to utilize a self-developed non-invasive, convenient real-time monitoring tool to achieve early screening and dynamic risk stratification for coronary heart disease (CHD) and its comorbidities through synchronous acquisition and analysis of PPW and ECG signals. The paper reports interim results of this research, which may provide valuable references for related fields. Subsequent work will focus on improving the acquisition device to enhance user comfort and prolong signal collection duration. Further multicenter data collection will be conducted to expand clinical sample size for model refinement. Additionally, in-depth comparisons with clinical risk scoring systems will be performed to clarify the advantages and limitations of this technology, with corresponding improvements made to provide multidimensional reference basis for clinical application.

## Data Availability

The raw data supporting the conclusions of this article will be made available by the authors, without undue reservation.
